# Supplementary material for: A Comprehensive Study from Cradle-to-Grave on the Environmental Profile of Malted Legumes
Source: Foods. 2024 Feb 21;13(5):655. doi: 10.3390/foods13050655 (PMC10930983; doi:10.3390/foods13050655)
Supplement: Supplementary file 1 [file foods-13-00655-s001.zip › foods-2863749-supplementary.pdf]

A comprehensive study from cradle-to-grave on the environmental profile of malted legumes

Mauro Moresi and Alessio Cimini

SUPPLEMENTARY MATERIAL S1

Table S1.1
Description of the product stages involved in the dried pulse life cycle network according to the Simapro software used in this work.

Product stage		Name	Main output	Unit	Ref. Table
Process	1	Pulse cultivation	Fresh pulse grains	kg	Table S1.2
Process	2	AlP production	AlP	kg	Table S1.3
Process	3	Fumigated pulse grains	Fumigated pulse grains	kg	Table S1.4
Process	4	1-Mg PE FIBC production	SS	kg	Table S1.5
Process	5	FIBC-packed cleaned dried pulse grains	Packed pulse grains	kg	Table S1.6
Process	6	Ready-to-pack dried pulse grains	Ready-to-pack pulse grains	kg	Table S1.7
Process	7	PP bag production	PP bags	kg	Table S1.8
Process	8	Cardboard collar production	Cardstocks	kg	Table S1.9
Process	9	Brass rivet production	Rivets	kg	Table S1.10
Process	10	Adhesive paper label production	Paper labels	kg	Table S1.11
Process	11	Carton production	Cartons	kg	Table S1.12
Process	12	Scotch tape production	Scotch tape	kg	Table S1.13
Process	13	PP semipallet production	PP semipallet	kg	Table S1.14
Process	14	Shrink & shrank PE film production	PE film	kg	Table S1.15
Assembly	15	Assembly of the primary packaging for dried pulses	Ass 1pack	p	Table S1.16
Assembly	16	Assembly of the secondary packaging for dried pulses	Ass 2pack	p	Table S1.17
Assembly	17	Assembly of the tertiary packaging for dried pulses	Ass 3pack	p	Table S1.18
Assembly	18	Assembly of the primary and secondary packages for dried pulses	Ass 1-2pack	p	Table S1.19
Assembly	19	Assembly of the primary, secondary and tertiary packages for dried pulses	Ass 1-2-3pack	p	Table S1.20
Assembly	20	Assembly of dried pulses	Ass dried pulses	p	Table S1.21
Processes	21	Use phase	Cooked pulses	kg	Table S1.22
Waste scenario	22	Waste scenario of plastic packaging wastes	PLW	kg	Table S1.23
Waste scenario	23	Waste scenario of paper and cardboard packaging wastes	PCW	kg	Table S1.24
Waste scenario	24	Waste scenario of non-ferrous metal wastes	NFMW	kg	Table S1.25
End of life scenario	25	Disposal scenario of primary packaging material wastes	DS-1pack	-	Table S1.26
End of life scenario	26	Disposal scenario of secondary packaging material wastes	DS-2pack	-	Table S1.27
End of life scenario	27	Disposal scenario of the tertiary packaging wastes	DS-3pack	-	Table S1.28
End of life scenario	28	Disposal scenario of the I, II, and III packaging material wastes	DS-1-2-3pack	-	Table S1.29
Reuse	29	Reuse of the PP semi-pallet	Pallet reuse	p	Table S1.30
Life cycle	30	Life cycle of dried pulses	LC-DP	p	Table S1.31
Life cycle	31	Life cycle of the primary, secondary and tertiary packaging materials	LC1-2-3pack	p	Table S1.32
Life cycle	32	Life cycle of the PP semi-pallet	LC-3pack	p	Table S1.33

Table S1.2
Inventory associated with the *Solco Dritto* chickpea cultivation phase.

Outputs to technosphere: Products and co-products	Amount	Unit	Quantit	Allocation %	Waste type	Category	Comment
SDC- 1 grains	CropYield*1000 = 1.71E3	kg	Mass	100 %	Biopolymers	Agricult...\Market	Solco Dritto Chickpeas -

Outputs to technosphere: Avoided products	Amount	Unit	Distribution	SD2 or 2SD	Min	Max	Comment

Inputs

Inputs from nature	Sub-compartment	Amount	Unit	Distribution	SD2 or 2SD	Min	Max	Comment
Occupation, annual crop, non-irrigated, intensive,		1	ha a	Undefined				
Water, river, IT		0	m3	Undefined				

Inputs from technosphere: materials/fuels	Amount	Unit	Distrit	SD2 (	Min	Max	Comment
NPK (15-15-15) fertiliser {RER}| market for NPK (15-15-15) fertiliser | Cut-off, S	NPKU*A = 133	kg					
Poultry manure, fresh {GLO}| market for poultry manure, fresh | Cut-off, S	PM*A = 200	kg					
Pesticide, unspecified {GLO}| market for pesticide, unspecified | Cut-off, S	DIS*A = 0.125	kg					
Lubricating oil {RER}| market for lubricating oil | Cut-off, S	LO*A = 1	kg					
Diesel combustion in agricultural machine {GLO} | diesel driven | production mix, at plant | 30kW to 180kW | LCI result	DF*DDF*A = 88.8	kg					PCI042.6 MJ/kG: engineeringtoolt
SDC - seeds	0	kg	Unde				
SDC - Chickpea seeding Mass, S	Seed*A = 130	kg					

Inputs from technosphere: electricity/heat	Amount
Transport, freight, light commercial vehicle {Europe without Switzerland}| market for transport, freight, light commercial vehicle | Cut-off, S	A*(Seed*25+NPKU*100+PM*100+DIS*25+DF*25+LO*25)/1000 = 39.2
Transport, tractor and trailer, agricultural {CH}| market for transport, tractor and trailer, agricultural | Cut-off, S	CropYield*DISTCA = 4.28

Outputs

Emissions to air	Sub-compartment	Amount	Unit	Distril	SD2 or	Min	Max	Comment
Dinitrogen monoxide		FN2O*A = 1.14	kg					
Nitrogen monoxide, IT		NO*A = 0.731	kg					
Carbon dioxide, fossil		0	kg	Unde				

Emissions to water	Sub-compartment	Amount	Unit	Distributio	SD2 or 2SI	Min	Max	Comment
Nitrate, IT	river, long-term	FNO3*A = 13.1	kg					
Phosphorus, IT	river, long-term	PLEACH*A = 0.611	kg					

Documentation | Input/output | **Parameters** | System description

Input parameters	Value	Distribution	SD2 or 2SD	Min	Max	Hide	Comment
CropYield	1.71	Triangle		1.11	2.35	☐	Resa granella fresca [Mg/(ha a)]
NPK	100	Triangle		90	110	☐	Fertilizzante NPK (20-20-20) [kg/ha]
PM	200	Triangle		180	220	☐	Poltry manure (4-4-4) [kg/ha]
DIS	0.125	Normal	0.05			☐	Herbicide [kg/ha]
DF	105	Triangle		90	120	☐	Diesel Fuel [L/ha]
LO	1	Undefined				☐	Lubricant Oil [L/ha]
A	1	Undefined				☐	Area [ha]
FRACrenew	1	Undefined				☐	Coltura annuale
RAG	2.1	Undefined				☐	Residui superficiali/Crop
FRACremoved	0	Undefined				☐	
FRACburned	0	Undefined				☐	
SSCrop	0.88	Undefined				☐	SS della granella raccolta [g/g]
NAG	0.008	Undefined				☐	Frazione azoto nella biomassa sup [g/g]
NBG	0.008	Undefined				☐	Frazione azoto nella biomassa radicale [g/g]
RBGBIO	0.19	Normal	0.07			☐	SS in BG/SS in AG [g/g]
Aburnt	0	Undefined				☐	Area ove si effettua l'incenerimento dei residui [ha]
Cf	0	Undefined				☐	Fattore di combustione
FRACgasf	0.11	Triangle		0.02	0.33	☐	kg N volatilizzato come NH3 e NOX/kg N sintetico applicato
FRACgasm	0.21	Triangle		0	0.31	☐	kg N volatilizzato come NH3 e NOX/kg N organico applicato
EF4	0.01	Triangle		0.002	0.018	☐	kg N2O-N/kg di NH3 e NOX volatilizzato
EF5	0.011	Triangle		0	0.02	☐	kg N2O-N/kg di N lisciviato o dilavato
FPRP	0	Undefined				☐	kg N nell'urina e sterco depositati da animali al pascolo [kg N/ha]

FSOM	0	Undefined				☐	kg N mineralizzato nei suoli ove si verifica perdita di C nella sostanza organica [kg N/ha]
FRACleach	0.24	Triangle		0.01	0.73	☐	kg N2O-N/kg di N lisciviato o dilavato/kg N applicato
EF1	0.01	Triangle		0.001	0.018	☐	kg N2O-N/kg N applicato
Seed	130	Triangle		120	140	☐	Densità di semina kg semi/ha]
DISTCA	2.5	Triangle		1	4	☐	Distanza campo-ammassatore [km]

Add

Calculated parameters	Expression	Comment
Crop	CropYield*SSCrop*1000 = 1.5E3	SS nella granella raccolta [kg/ha]
AG	(Crop/1000*1.13+.85)*1000 = 2.55E3	Sostanza secca nella biomassa sup [kg/h]
RBG	RBGBIO*(AG+Crop)/Crop = 0.512	Rapporto fra la SS nei residui radicali/SS nella biomassa raccolta g/g]
BG	RBG*Crop = 770	SS nei residui sotterranei [kg/ha]
FCR	FRACrenew*((A-Aburnt*Cf)*AG*NAG*(1-FRACremoved)+A*BG*NBG) = 26.6	N nei residui sup e sott restituiti al suolo [kg/ha]
NPKU	NPK*20/15 = 133	Fertilizzante NPK (15-15-15) [kg/ha]
FSN	0.15*NPKU = 20	N sintetico [kg/ha]
FON	0.04*PM = 8	N organico [kg/ha]
FNH3NOX	FSN*FRACgasf+(FON+FPRP)*FRACgasm = 3.88	kg NH3 e NOX volatilizzato/ha
FNO3	(FSN+FON+FPRP+FCR+FSOM)*FRACleach = 13.1	kg N lisciviato o dilavato/ha
FN2OD	44/28*A*(FSN+FON+FCR+FSOM)*EF1 = 0.857	kg N2O-N dirette
FN2OIND	44/28*A*(FNH3NOX*EF4+FNO3*EF5) = 0.287	kg N2O-N indirette
FN2O	FN2OD+FN2OIND = 1.14	kg N2O-N totali
NOAN	0.5*NPK/(2*14+4+3*16)*(2*14) = 17.5	kg N/ha se 50% NPK = AN (NH4NO3)
NOCAN	0.5*NPK*(4*14)/(40+4+4*14+9*16) = 11.5	kg N/ha se 50% NPK = CAN (Ca NH4 (NO3)3)
NO	NOAN*0.029+NOCAN*0.016+FON*0.005 = 0.731	EPD (2020) Emissioni NO [kg/ha]
PLEACH	(NPKU*.15+PM*.04)*(2*31)/(2*31+5*16)*.05 = 0.611	P lisciviato= 0.05 P sint ed organico [kg/ha]

Table S1.3
Inventory associated with the production of Aluminum phosphide (AlP) tablets.

Products

Outputs to technosphere: Products and co-products	Amount	Unit	Quantit	Allocation %	Waste type	Category	Comment
SDC - 3 AlP	ALPTABLET = 1	kg	Mass	100 %	Aluminium	Chemicals	

Outputs to technosphere: Avoided products	Amount	Unit	Distribution	SD2 or 2SD	Min	Max	Comment

Inputs

Inputs from nature	Sub-compartment	Amount	Unit	Distribution	SD2 or 2SD	Min	Max	Comment

Inputs from technosphere: materials/fuels	Amount	Unit	Distrit	SD2 (	Min	Max	Comment
Aluminium, primary, liquid {GLO}| market for aluminium, primary, liquid | Cut-off, S	ALPTABLET*0.46552*xAlP = 0.261	kg					
Phosphorus, white, liquid {GLO}| market for phosphorus, white, liquid | Cut-off, S	ALPTABLET*0.53448*xAlP = 0.299	kg					
Aluminium oxide, non-metallurgical {IAI Area, Russia & RER w/o EU27 & EFTA}| aluminium ox	ALPTABLET*(xAlO+xAlS) = 0.17	kg					
Graphite {GLO}| market for graphite | Cut-off, S	ALPTABLET*xG = 0.015	kg					
Urea {RER}| urea production | Cut-off, S	ALPTABLET*xU = 0.18	kg					
Ammonium carbonate {RER}| market for ammonium carbonate | Cut-off, S	ALPTABLET*xAMC = 0.05	kg					
Paraffin {GLO}| market for paraffin | Cut-off, S	ALPTABLET*xPW = 0.025	kg					

Inputs from technosphere: electricity/heat	Amount	Unit	Dis	S	Comment
Heat, district or industrial, natural gas {Europe without Switzerland}| market for heat, district or industrial, natural gas | Cut-off, S	ALPTABLET*xAlP*(0.46552*En	kJ			
Dolomite grinding {GLO} | dolomite grinding | production mix, at plant | 2.90 g/cm3 | Partly terminated system	ALPTABLET*xAlP = 0.56	kg			Grinding of P and Al
Transport, freight, lorry 3.5-7.5 metric ton, EURO5 {RER}| transport, freight, lorry 3.5-7.5 metric ton, EURO5 | Cut-off, S	ALPTABLET/1000*300 = 0.3	tkm			TR: MP - Fondi: 200 km
Transport, freight, light commercial vehicle {Europe without Switzerland}| transport, freight, light commercial vehicle | Cut-off, S	ALPTABLET/1000*280 = 0.28	tkm			TR: Fondi-AP: 280 km

Documentation | Input/output | **Parameters** | System description

Input parameters	Value	Distribution	SD2 or 2SD	Min	Max	Hide	Comment
xAIP	0.56	Undefined				☐	Frazione di AIP nelle tablets
xAMC	0.05	Undefined				☐	Frazione di ammonium carbonate (w/w)
xAIS	0.025	Undefined				☐	Frazione di aluminium stearate (w/w)
xAIO	0.145	Undefined				☐	Frazione di aluminium oxide/hydroxide (w/w)
xU	0.18	Undefined				☐	Frazione di urea (w/w)
xG	0.015	Undefined				☐	Frazione di graphite (w/w)
xPW	0.025	Undefined				☐	Frazione di paraffin wax (w/w)
ALPTABLET	1	Undefined				☐	Massa di una compressa [g]
CpPS	0.77	Undefined				☐	Specifi heat value of solid P {kJ/(kg K)]: https://www.periodic-table.org/phosphorus-latent-heat-vaporization/
CpPL	0.85	Undefined				☐	Specific heat value of liquid P {kJ/(kg K)]: https://webbook.nist.gov/cgi/inchi?ID=C7723140&Mask=2
CpPV	0.5	Undefined				☐	Specific heat value of vapor P {kJ/(kg K)]
DELTAHfusP	0.657	Undefined				☐	[kJ/mol]
DELTAHvapP	51.9	Undefined				☐	[kJ/mol]
CpAIS	0.9	Undefined				☐	Specifi heat value of solid Al {kJ/(kg K)]
CpAIL	1.127	Undefined				☐	Specifi heat value of liquid Al {kJ/(kg K)]
DELTAHfusAl	10.79	Undefined				☐	[kJ/mol]
TfusP	44.1	Undefined				☐	Temperatura di fusione P {°C]
TBP	280	Undefined				☐	Temperatura di ebollizione P °C
TfusAL	660	Undefined				☐	Temperatura di fusione Al {°C]
TR	700	Undefined				☐	Temperatura di reazione °C]

Calculated parameters	Expression	Comment
EntP	CpPS*(TfusP-20)+DELTAHfusP/0.031+CpPL*(TBP-TfusP)+DELTAHvapP/.031+CpPV*(TR-TBP) = 2.1	Calore di riscald P [kJ/kg]
EntAl	CpAIS*(TfusAL-20)+DELTAHfusAl/0.027+CpAIL*(TR-TfusAL) = 1.02E3	Calore di riscald Al [kJ/kg]

Table S1.4

Inventory associated with the pulse grain fumigation step.

Products

Outputs to technosphere: Products and co-products	Amount	Unit	Quantit	Allocation %	Waste type	Category	Comment
SDC - 4 Fumigated grains	17100.171	kg	Mass	100 %	Biopolymers	Agr...\Plant seeds	

Outputs to technosphere: Avoided products	Amount	Unit	Distribution	SD2 or 2SD	Min	Max	Comment

Inputs

Inputs from nature	Sub-compartment	Amount	Unit	Distribution	SD2 or 2SD	Min	Max	Comment

Inputs from technosphere: materials/fuels	Amount	Unit	Distrit	SD2 (	Min	Max	Comment
SDC- 1 grains	17100	kg	Unde				
SDC - 3 AIP	0.2565	kg	Unde				

Inputs from technosphere: electricity/heat	Amount	Unit	Dis	S	Comment

Outputs

Emissions to air	Sub-compartment	Amount	Unit	Distril	SD2 or	Min	Max	Comment
Phosphine		85.5	g	Unde				

Documentation | Input/output | **Parameters** | System description

Input parameters	Value	Distribution	SD2 or 2SD	Min	Max	Hide	Comment
SCPUL	12.5	Triangle		10	15	☐	Scarto di pulitura [%]
DUST	2	Triangle		1.6	2.4	☐	Polvere [%}
GI	6	Triangle		4.8	7.2	☐	Grass & Insects [%]
WASTE	4.5	Triangle		3.6	5.4	☐	Legumi spezzati etc [%]

Calculated parameters	Expression	Comment

Table S1.5

Inventory associated with the production of 1-Mg PE bags.

Products

Outputs to technosphere: Products and co-products	Amount	Unit	Quantit	Allocation %	Waste type	Category	Comment
SDC - 2 PE Supersacks	3	kg	Mass	100 %	LDPE	P...\Thermoplasts	

Outputs to technosphere: Avoided products	Amount	Unit	Distribution	SD2 or 2SD	Min	Max	Comment

Inputs

Inputs from nature	Sub-compartment	Amount	Unit	Distribution	SD2 or 2SD	Min	Max	Comment

Inputs from technosphere: materials/fuels	Amount	Unit	Distrib	SD2 (	Min	Max	Comment
Packaging film, low density polyethylene {RER}| packaging film production, low density polye	3/0.976 = 3.07	kg					Resa di conversione in film: 0.976 kg/kg P

Inputs from technosphere: electricity/heat	Amount	Unit	Dis	S	Comment
Extrusion, plastic film {RER}| extrusion, plastic film | Cut-off, S	3/.976 = 3.07	kg			0.976 kg
Transport, freight, light commercial vehicle {Europe without Switzerland}| transport, freight, light commercial vehicle | Cut-off, S	3/1000*355 = 1.06	tkm			Distanza SS - Azienda conf: 355 km
Transport, freight, lorry 16-32 metric ton, EURO5 {RER}| transport, freight, lorry 16-32 metric ton, EURO5 | Cut-off, S	(3/.976)/1000*300 = 0.922	tkm			Distanza Fornitore PE - Azienda RE: 300 km

Outputs to technosphere: Waste and emissions to treatment	Amount	Unit	Distri
Waste polyethylene {Europe without Switzerland}| market group for waste polyethylene | Cut-off, S	3*(1-0.976) = 0.072	kg	

Table S1.6

Inventory associated with the flexible intermediate bulk container (FIBC)-packed cleaned dry pulse grain production step.

Products

Outputs to technosphere: Products and co-products	Amount	Unit	Quantit	Allocation %	Waste type	Category	Comment
SDC - 5 FIBC-packed cleaned dry pulse grains	GPP = 1.5E4	kg	Mass	99.6 %	Biopolymers	Agr...\Plant seeds	Legumi puliti nei supersacch
SDC - waste	2137.7	kg	Mass	0.4 %	Biopolymers	Agr...\Plant seeds	
Add							

Outputs to technosphere: Avoided products	Amount	Unit	Distribution	SD2 or 2SD	Min	Max	Comment
Add							

Inputs

Inputs from nature	Sub-compartment	Amount	Unit	Distribution	SD2 or 2SD	Min	Max	Comment
Add								

Inputs from technosphere: materials/fuels	Amount	Unit	Distrit	SD2 (	Min	Max	Comment
SDC - 4 Fumigated grains	17100.171	kg	Unde				
SDC - 2 PE Supersacks	44.9	kg	Unde				
Add							

Inputs from technosphere: electricity/heat	Amount	Unit	Dis	S	Comment
Add					

Table S1.7
Inventory associated with the production of ready-to-pack dried pulses.

Documentation | Input/output | **Parameters** | System description

Input parameters	Value	Distribution	SD2 or 2SD	Min	Max	Hide	Comment
GP	14962.5	Undefined				☐	Cleaned pulses grains [kg]
SS	44.9	Undefined				☐	PE-sacks [kg]
Add							

Calculated parameters	Expression	Comment
GPP	GP+SS = 1.5E4	PE-bagged cleaned pulse grains [kg]
Add		

Products

Outputs to technosphere: Products and co-products	Amount	Unit	Quantit	Allocation %	Waste type	Category	Comment
SDC - 6 ready-to-pack dry grains	GPD = 1.31E4	kg	Mass	100 %	Biopolymers	Agr...\Plant seeds	Legumi puliti nei supersacch
SDC - DGI	GRF*(DUST+GI) = 1.37E3	kg	Mass	0 %	Others	Others	Dust, grass & Insect waste
SDC - MZ	GRF*BP+0.005*GPD = 835	kg	Mass	0 %	Biopolymers	Ag...\Bovine feed	Pulse Waste used as cattle fe
SDC - seeds	Seed*A = 1.3E3	kg	Mass	0 %	Biopolymers	Agr...\Plant seeds	Seeds used
Add							

Outputs to technosphere: Avoided products	Amount	Unit	Distribution	SD2 or 2SD	Min	Max	Comment
Add							

Inputs

Inputs from nature	Sub-compartment	Amount	Unit	Distribution	SD2 or 2SD	Min	Max	Comment
Add								

Inputs from technosphere: materials/fuels	Amount	Unit	Distrit	SD2 (	Min	Max	Comment
SDC - 5 FIBC-packed cleaned dry pulse grains	GPP = 1.5E4	kg					
Add							

Inputs from technosphere: electricity/heat	Amount	Unit	Dis	S	Comment
Transport, tractor and trailer, agricultural {RoW}| transport, tractor and trailer, agricultural | Cut-off, S	GRF*(DUST+GI)/1000*4 = 5.4	tkm			TR: AP- field: 4 km
Transport, tractor and trailer, agricultural {RoW}| transport, tractor and trailer, agricultural | Cut-off, S	(GRF*BP+0.005*GPD)/1000*(1	tkm			TR: AP - Beef cattle farms: 50 km

Outputs to technosphere: Waste and emissions to treatment	Amount	Unit	Distri
Waste polyethylene {RoW}| treatment of waste polyethylene, municipal incineration | Cut-off, S	SS*0.446 = 20	kg	
Waste polyethylene {RoW}| treatment of waste polyethylene, sanitary landfill | Cut-off, S	SS*0.067 = 3.01	kg	
PE (waste treatment) {GLO}| recycling of PE | Cut-off, S	SS*0.487 = 21.9	kg	

Table S1.8

Inventory associated with the PP bag production step.

Products

Outputs to technosphere: Products and co-products	Amount	Unit	Quantit	Allocation %	Waste type	Category	Comment
SDC- 7 PP bags	1	kg	Mass	100 %	PP	P...\Thermoplasts	

Outputs to technosphere: Avoided products	Amount	Unit	Distribution	SD2 or 2SD	Min	Max	Comment

Inputs

Inputs from nature	Sub-compartment	Amount	Unit	Distribution	SD2 or 2SD	Min	Max	Comment

Inputs from technosphere: materials/fuels	Amount	Unit	Distrit	SD2 (	Min	Max	Comment
Polypropylene, granulate {GLO}| market for polypropylene, granulate | Cut-off, S	1/.976 = 1.02	kg					Resa: 0.976 kg /kg PP

Inputs from technosphere: electricity/heat	Amount	Unit	Dis	S	Comment
Extrusion, plastic film {RER}| extrusion, plastic film | Cut-off, S	1/.976 = 1.02	kg			
Transport, freight, lorry 16-32 metric ton, EURO5 {RER}| transport, freight, lorry 16-32 metric ton, EURO5 | Cut-off, S	(1/.976)/1000*300 = 0.307	tkm			TR PP Granules Prod Site - Pl: 300 km

Outputs to technosphere: Waste and emissions to treatment	Amount	Unit	Distri
Waste polypropylene {Europe without Switzerland}| market group for waste polypropylene | Cut-off, S	0.024	kg	Unde

Table S1.9
Inventory associated with the cardboard collar production step.

Products

Outputs to technosphere: Products and co-products	Amount	Unit	Quantit	Allocation %	Waste type	Category	Comment
SDC - 8 Cardstock	1	kg	Mass	100 %	not defined	Agr...\Plant seeds	
Add							

Outputs to technosphere: Avoided products	Amount	Unit	Distribution	SD2 or 2SD	Min	Max	Comment
Add							

Inputs

Inputs from nature	Sub-compartment	Amount	Unit	Distribution	SD2 or 2SD	Min	Max	Comment
Add								

Inputs from technosphere: materials/fuels	Amount	Unit	Distrit	SD2 ‹	Min	Max	Comment
Kraft paper {RER}| market for kraft paper | Cut-off, S	1	kg	Unde				
Add							

Inputs from technosphere: electricity/heat	Amount	Unit	Dis	S	Comment
Printed paper, offset {RoW}| offset printing, per kg printed paper | Cut-off, S	1	kg	Un		
Transport, freight, lorry >32 metric ton, EURO5 {RER}| transport, freight, lorry >32 metric ton, EURO5 | Cut-off, S	(1/1000)*300 = 0.3	tkm			TR Cartoncino PS-Montefiascone: 300 km

Table S1.10
Inventory associated with the brass rivet production step.

Products							
Outputs to technosphere: Products and co-products	Amount	Unit	Quantit	Allocation %	Waste type	Category	Comment
SDC - 9 - Brass rivets	1	kg	Mass	100 %	Non-ferro	Metals\Non Ferrc	

Outputs to technosphere: Avoided products	Amount	Unit	Distribution	SD2 or 2SD	Min	Max	Comment

Inputs								
Inputs from nature	Sub-compartment	Amount	Unit	Distribution	SD2 or 2SD	Min	Max	Comment

Inputs from technosphere: materials/fuels	Amount	Unit	Distrit	SD2 (	Min	Max	Comment
Brass {RoW}| market for brass | Cut-off, S	1	kg	Unde				

Inputs from technosphere: electricity/heat	Amount	Unit	Dis	S			Comment
Brass Die-Casting {GLO} | die casting, from copper and zinc, primary production | production mix, at plant | 8.41- 8.86 g/cm3 | Pai	1	kg	Un				
Transport, freight, lorry 16-32 metric ton, EURO5 {RER}| transport, freight, lorry 16-32 metric ton, EURO5 | Cut-off, S	(1/1000)*300 = 0.3	tkm					TR Ottone: PS- PI: 300 km

Table S1.11
Inventory associated with the adhesive paper label production step.

Products

Outputs to technosphere: Products and co-products	Amount	Unit	Quantit	Allocation %	Waste type	Category	Comment
SDC - 10 Adhesive paper labels	1	kg	Mass	100 %	Paper	...\Graphic paper	
Add							

Outputs to technosphere: Avoided products	Amount	Unit	Distribution	SD2 or 2SD	Min	Max	Comment
Add							

Inputs

Inputs from nature	Sub-compartment	Amount	Unit	Distribution	SD2 or 2SD	Min	Max	Comment
Add								

Inputs from technosphere: materials/fuels	Amount	Unit	Distrit	SD2 (	Min	Max	Comment
Graphic paper, 100% recycled {GLO}| market for graphic paper, 100% recycled | Cut-off, S	1	kg	Unde				
Add							

Inputs from technosphere: electricity/heat	Amount	Unit	Dis	S	Comment
Transport, freight, lorry 16-32 metric ton, EURO5 {RER}| market for transport, freight, lorry 16-32 metric ton, EURO5 | Cut-off, S	(1/1000)*300 = 0.3	tkm			TR Paper x Labels: PS -Montefiascone: 300 km
Acrylic binder, with water, in 54% solution state {RER}| market for acrylic binder, with water, in 54% solution state | Cut-off, S	5*1210/(5*1210+600*1000) =	kg			

Table S1.12

Inventory associated with the carton production step (copied from Carton box {EU+EFTA+UK} | Kraft Pulping Process, pulp pressing and drying, box manufacturing | production mix, at plant | 280 g/m², R1=47% | LCI result, EF database 3.1 Corepackage – Sphera).

Products

Outputs to technosphere: Products and co-products	Amount	Unit	Quantit	Allocation %	Waste type	Category	Comment
SDC - 11 Carton box	1.0	kg	Mass	100 %	Cardboard	Pap...\Packaging	Product flow name: Carton b
Add							

Outputs to technosphere: Avoided products	Amount	Unit	Distribution	SD2 or 2SD	Min	Max	Comment
Add							

Inputs

Inputs from nature	Sub-compartment	Amount	Unit	Distribution	SD2 or 2SD	Min	Max	Comment
Air	in air	5.1649277E1	kg	Undefined				

Inputs from technosphere: electricity/heat	Amount	Unit	Dis	S	Comment
Transport, freight, lorry 16-32 metric ton, EURO5 {RER}| transport, freight, lorry 16-32 metric ton, EURO5 | Cut-off, S	(1/1000)*300 = 0.3	tkm			TR PS-PG: 300 km

Table S1.13
Inventory associated with the scotch tape production step.

Products

Outputs to technosphere: Products and co-products	Amount	Unit	Quantit	Allocation %	Waste type	Category	Comment
SDC- 12 Scotch	1	kg	Mass	100 %	PP	P...\Thermoplasts	
Add							

Outputs to technosphere: Avoided products	Amount	Unit	Distribution	SD2 or 2SD	Min	Max	Comment
Add							

Inputs

Inputs from nature	Sub-compartment	Amount	Unit	Distribution	SD2 or 2SD	Min	Max	Comment
Add								

Inputs from technosphere: materials/fuels	Amount	Unit	Distrit	SD2 (	Min	Max	Comment
Polypropylene, granulate {GLO}| market for polypropylene, granulate | Cut-off, S	(1-0.0425)/.976 = 0.981	kg					Resa: 0.976 kg /kg PP
Add							

Inputs from technosphere: electricity/heat	Amount	Unit	Dis	S	Comment
Extrusion, plastic film {RER}| extrusion, plastic film | Cut-off, S	(1-0.0425)/.976 = 0.981	kg			
Transport, freight, lorry 7.5-16 metric ton, EURO5 {RER}| transport, freight, lorry 7.5-16 metric ton, EURO5 | Cut-off, S	((1-0.0425)/.976+0.0425)/100(	tkm			TR PP Granules Prod Site - PI: 300 km
Acrylic binder, with water, in 54% solution state {RER}| market for acrylic binder, with water, in 54% solution state | Cut-off, S	10*1210/(10*1210+300*950) :	kg			tape thickness 300 micron (950 kg/m3), acrylic layer 10 micron (1210 kg/m3)

Outputs to technosphere: Waste and emissions to treatment	Amount	Unit	Distri
Waste polypropylene {Europe without Switzerland}| market group for waste polypropylene | Cut-off, S	0.024	kg	Unde

Table S1.14
Inventory associated with the PP semi-pallet production step.

Products

Outputs to technosphere: Products and co-products	Amount	Unit	Quantit	Allocation %	Waste type	Category	Comment
SDC - 13 semipallet	1	kg	Mass	100 %	PP	P...\Thermoplasts	
Add							

Outputs to technosphere: Avoided products	Amount	Unit	Distribution	SD2 or 2SD	Min	Max	Comment
Add							

Inputs

Inputs from nature	Sub-compartment	Amount	Unit	Distribution	SD2 or 2SD	Min	Max	Comment
Add								

Inputs from technosphere: materials/fuels	Amount	Unit	Distrit	SD2 (	Min	Max	Comment
Polypropylene, recycled, pre-consumer {GLO} | shredding, pelletizing | production mix, at plan	1/.98 = 1.02	kg					Resa: 0.98 kg /kg PP
Add							

Inputs from technosphere: electricity/heat	Amount	Unit	Dis	S	Comment
Injection moulding {RER}| injection moulding | Cut-off, S	1/.98 = 1.02	kg			
Transport, freight, lorry 16-32 metric ton, EURO5 {RoW}| transport, freight, lorry 16-32 metric ton, EURO5 | Cut-off, S	(1/.98)/1000*300 = 0.306	tkm			TR PP Granules Prod Site - Pl: 300 km

Outputs to technosphere: Waste and emissions to treatment	Amount	Unit	Distri
Waste polypropylene {Europe without Switzerland}| market group for waste polypropylene | Cut-off, S	0.02	kg	Unde

Table S1.15
Inventory associated with the shrink & shrank PE film production step.

Products							
Outputs to technosphere: Products and co-products	Amount	Unit	Quantit	Allocation %	Waste type	Category	Comment
SDC- 14 PE shrinkable film	1	kg	Mass	100 %	PE	P...\Thermoplasts	
Add							

Outputs to technosphere: Avoided products	Amount	Unit	Distribution	SD2 or 2SD	Min	Max	Comment
Add							

Inputs								
Inputs from nature	Sub-compartment	Amount	Unit	Distribution	SD2 or 2SD	Min	Max	Comment
Add								

Inputs from technosphere: materials/fuels	Amount	Unit	Distrit	SD2 (	Min	Max	Comment
Polyethylene, low density, granulate {RER}| polyethylene production, low density, granulate | C	1/.976 = 1.02	kg					Resa: 0.976 kg /kg PP
Add							

Inputs from technosphere: electricity/heat	Amount	Unit	Dis	S	Comment
Extrusion, plastic film {RER}| extrusion, plastic film | Cut-off, S	1/.976 = 1.02	kg			
Transport, freight, lorry 16-32 metric ton, EURO5 {RER}| transport, freight, lorry 16-32 metric ton, EURO5 | Cut-off, S	(1/.976)/1000*300 = 0.307	tkm			TR PP Granules Prod Site - PI: 300 km

Outputs to technosphere: Waste and emissions to treatment	Amount	Unit	Distri
Waste polyethylene {ES}| market for waste polyethylene | Cut-off, S	0.024	kg	Unde

Table S1.16
Assembly of the primary packaging for dried pulses.

Input/output | Parameters

Name	Status	Comment
SDC - 15 Assembly of Primary Packaging	None	

Materials/Assemblies	Amount	Unit	Distribution	SD2 or 2SD	Min	Max	Comment
SDC- 7 PP bags	PPBAG = 5	g					
SDC - 8 Cardstock	CAV = 5.8	g					
SDC - 9 - Brass rivets	2*RIV = 0.66	g					
SDC - 10 Adhesive paper labels	ET = 0.323	g					
Add							

Processes	Amount	Unit	D	SD:	M	M	Comment
Transport, freight, lorry 7.5-16 metric ton, EURO5 {RER}| market for transport, freight, lorry 7.5-16 metric ton, EURO5 | Cut-off, S	(PPBAG+2*RIV)/1000*200 = 1.13	kgkm					TR: PI-AP: 200 km
Transport, freight, light commercial vehicle {Europe without Switzerland}| market for transport, freight, light commercial vehicle | Cut-off, S	(CAV+ET)/1000*30 = 0.184	kgkm					TR: Montefiascone-AP: 30 km

Input/output | **Parameters**

Input parameters	Value	Distribution	SD2 or 2SD	Min	Max	Hide	Comment
PPBAG	5	Normal	0.3			☐	
CAV	5.8	Normal	0.6			☐	
RIV	0.33	Normal	0.03			☐	
ET	0.323	Normal	0.03			☐	

Table S1.17
Assembly of the secondary packaging for dried pulses.

Input/output | Parameters

Name	Status	Comment
SDC - 16 Assembly of Secondary Packaging	None	

Materials/Assemblies	Amount	Unit	Distribution	SD2 or 2SD	Min	Max	Comment
SDC - 11 Carton box	CA = 302	g					
SDC - 10 Adhesive paper labels	EC = 2	g					
SDC- 12 Scotch	SC = 4	g					

Add

Processes	Amount	Unit	D	SD:	M	M	Comment
Transport, freight, light commercial vehicle {Europe without Switzerland}| market for transport, freight, light commercial vehicle | Cut-off, S	EC/1000*30 = 0.06	kgkm					TR: Montefiascone - AP: 30 km
Transport, freight, lorry 7.5-16 metric ton, EURO5 {RER}| market for transport, freight, lorry 7.5-16 metric ton, EURO5 | Cut-off, S	(CA*105+SC*200)/1000 = 32.5	kgkm					TR: PG o PI - AP: 105 o 200 km

Input/output | **Parameters**

Input parameters	Value	Distribution	SD2 or 2SD	Min	Max	Hide	Comment
CA	302	Normal	3			☐	g
EC	2.0	Normal	0.2			☐	g
SC	4	Normal	0.4			☐	g

Add

Table S1.18

Assembly of the tertiary packaging for dried pulses.

Input/output | Parameters

Name	Status	Comment
SDC - 17 Assembly of Tertiary Packaging	None	

Materials/Assemblies	Amount	Unit	Distribution	SD2 or 2SD	Min	Max	Comment
SDC - 13 semipallet	PAL = 5	kg					
Add							

Processes	Amount	Unit	D	M	M	Comment
Transport, freight, lorry 7.5-16 metric ton, EURO5 {RER}| market for transport, freight, lorry 7.5-16 metric ton, EURO5 | Cut-off, S	(PAL*0.02)*200 = 20	kgkm				TR: PI - AP: 200 km (solo frazione da reintragrare: 0.02%

Input/output | **Parameters**

Input parameters	Value	Distribution	SD2 or 2SD	Min	Max	Hide	Comment
PAL	5	Normal	0.1			☐	kg
Add							

Table S1.19
Assembly of primary and secondary packages for dried pulses.

Input/output | Parameters

Name	Status	Comment
SDC - 18 Assembly of I-II packages	None	

Materials/Assemblies	Amount	Unit	Distribution	SD2 or 2SD	Min	Max	Comment
SDC - 15 Assembly of Primary Packaging	12	p	Undefined				
SDC - 16 Assembly of Secondary Packaging	1	p	Undefined				
Add							

Processes	Amount	Unit	D	M	M	Comment
Add						

Table S1.20
Assembly of primary, secondary, and tertiary packages for dried pulses.

Input/output | Parameters

Name	Status	Comment
SDC - 19 Assembly of I-II-III packages	None	

Materials/Assemblies	Amount	Unit	Distribution	SD2 or 2SD	Min	Max	Comment
SDC - 18 Assembly of I-II packages	24	p	Undefined				
SDC- 14 PE shrinkable film	FP = 287	g					
SDC - 10 Adhesive paper labels	EP*2 = 6.22	g					
SDC - 17 Assembly of Tertiary Packaging	1	p	Undefined				

Add

Processes	Amount	Unit	D	M	M	Comment
Transport, freight, lorry 7.5-16 metric ton, EURO5 {RER}| market for transport, freight, lorry 7.5-16 metric ton, EURO5 | Cut-off, S	(FP/1000)*200 = 57.4	kgkm				TR: PI - AP: 200 km
Transport, freight, light commercial vehicle {Europe without Switzerland}| market for transport, freight, light commercial vehicle | C	(EP*2/1000)*30 = 0.187	kgkm				TR: Montefiascone - AP: 30 km

Input/output | **Parameters**

Input parameters	Value	Distribution	SD2 or 2SD	Min	Max	Hide	Comment
EP	3.11	Normal	0.3			☐	g
FP	287	Normal	20			☐	g

Table S1.21
Assembly of dried pulses (1 Functional Unit).

Input/output | Parameters

Name	Status	Comment
SDC - 20 Assembly of Dry Grains	None	

Materials/Assemblies	Amount	Unit	Distribution	SD2 or 2SD	Min	Max	Comment
SDC - 5 FIBC-packed cleaned dry pulse grains	MDP = 0.5	kg					
Nitrogen gas production {EU+EFTA+UK} | technology mix | production mix, at plant | 100% active substance | LCI result	N2 = 5	g					
Add							

Processes	Amount	Unit	D	M	M	Comment
Transport, freight, lorry 7.5-16 metric ton, EURO5 {RER}| market for transport, freight, lorry 7.5-16 metric ton, EURO5 | Cut-off, S	N2/1000*35 = 0.175	kgkm				TR: Orvieto-AP: 35 km
Electricity, medium voltage {IT}| market for electricity, medium voltage | Cut-off, S	EECONS*MDP = 0.33	kWh				EE consumata

Input/output | **Parameters**

Input parameters	Value	Distribution	SD2 or 2SD	Min	Max	Hide	Comment
MDP	0.5	Undefined				☐	Dried pulse mass kg]
N2	5	Normal	0.5			☐	Nitrogen consumed per 0.5-kg dry pulse bag [g]
EECONS	0.66	Undefined				☐	Electricity consumed [kWh/kg dry pulses]

Table S1.22
Inventory associated with the use phase.

Products

Outputs to technosphere: Products and co-products	Amount	Unit	Quantit	Allocation %	Category	Comment
SDC - 21 Use phase	RDP = 1	kg	Mass	100 %	Others	
SDC - Cooked pulses wasted	MPC*CPWASTED = 0.262	kg	Mass	0 %	Others	
Add						

Outputs to technosphere: Avoided products	Amount	Unit	Distribution	SD2 or 2SD	Min	Max	Comment
Add							

Inputs

Inputs from nature	Sub-compartment	Amount	Unit	Distribution	SD2 or 2SD	Min	Max	Comment
Add								

Inputs from technosphere: materials/fuels	Amount	Unit	Distrit	SD2 c	Min	Max	Comment
Tap water {Europe without Switzerland}| market for tap water | Cut-off, S	(WAM+WAC)*RDP+CONSW*NCL = 15.8	kg					Acqua per amm+ cott + acqua x lavastov
Detergent dish production {EU+EFTA+UK} | production mix | 1 kg of detergent dish | LCI resul	CONSDET*NCL = 7.14	g					Detersivo consumato x lavastoviglie
Add							

Inputs from technosphere: electricity/heat	Amount	Unit	Dis	S	Comment
Heat, central or small-scale, natural gas {Europe without Switzerland}| market for heat, central or small-scale, natural gas | Cut-of	ECG*RDP = 1.41	kWh			Consumo Gas nat fornelli a gas
Electricity, low voltage {IT}| market for electricity, low voltage | Cut-off, S	ECE*RDP+CONSEE*NCL = 0.8	kWh			Consumo EE (fornello+lavastoviglie)
Municipal waste collection service by 21 metric ton lorry {GLO}| market for municipal waste collection service by 21 metric ton lo	MPC*CPWASTED*50 = 13.1	kgkm			TR: House-MSWCS: 50 km

Outputs

Emissions to air	Sub-compartment	Amount	Unit	Distril	SD2 or	Min	Max	Comment
Water (evapotranspiration)	low. pop.	WAC*RDP*Wevap = 0.2	kg					
Nitrogen, total	low. pop.	10*RDP = 10	g					

Outputs to technosphere: Waste and emissions to treatment	Amount	Unit	Distri
Sludge from pulp and paper production {Europe without Switzerland}| treatment of sludge from pulp and paper production, sanitary landfill | Cut-off, S	MPC*CPWASTED*.31 = 0.0812	kg	
Raw sewage sludge {RoW}| treatment of raw sewage sludge, municipal incineration | Cut-off, S	MPC*CPWASTED*.18 = 0.0472	kg	
Biowaste {RoW}| treatment of biowaste by anaerobic digestion | Cut-off, S	MPC*CPWASTED*.255 = 0.0668	kg	
Biowaste {RoW}| treatment of biowaste, industrial composting | Cut-off, S	MPC*CPWASTED*.255 = 0.0668	kg	
Wastewater, average {Europe without Switzerland}| market for wastewater, average | Cut-off, S	(WASTEWAM+WASTEWC)*RDP+CONSW*NCL = 14	l	

Documentation | Input/output | **Parameters** | System description

Input parameters	Value	Distribution	SD2 or 2SD	Min	Max	Hide	Comment
RDP	1	Undefined				☐	Dry pulses [kg]
XMDP	0.083	Undefined				☐	Umidità iniziale legumi [g/g]
WAM	4	Undefined				☐	Acqua x ammollo [L/kg dried pulses]
XMAM	0.55	Triangle		0.5	0.6	☐	Umidità legumi post-ammollo [g/g]
WAC	4	Undefined				☐	Acqua x cottura [L/kg dried pulses]
XMC	0.65	Triangle		0.62	0.68	☐	Umidità legumi post-cottura [g/g]
GCH	0.83	Undefined				☐	Frazione dei fornelli a gas
ECH	0.17	Undefined				☐	Frazione dei fornelli elettrici
ETC	1.7	Undefined				☐	Energia totale di cottura [kWh/kg]
CPWASTED	0.1	Triangle		0.08	0.12	☐	Frazione legumi cotti sprecati [g/g]
Portion	50	Undefined				☐	Porzione consumata di legumi secchi [g]
NCOP	28	Undefined				☐	No coperti/ciclo di lavaggio
CONSEE	0.74	Undefined				☐	Cons EE [kWh/ciclo di lavaggio]
CONSW	10.9	Undefined				☐	Cons acqua [L/ciclo di lavaggio]
CONSDET	10	Undefined				☐	Cons detersivo [g/ciclo di lavaggio]
Wevap	0.05	Undefined				☐	Frazione acqua di cottura evaporata [g/g]

Add

Calculated parameters	Expression	Comment
MPI	RDP*(1-XMDP)/(1-XMAM) = 2.04	Massa legumi post-ammollo [kg]
WASTEWAM	WAM*RDP - (MPI*XMAM-RDP*XMDP) = 2.96	Acqua da smaltire post-ammollo [kg]
MPC	RDP*(1-XMDP)/(1-XMC) = 2.62	Massa legumi post-cottura [kg]
WASTEWC	WAC*RDP-(MPC*XMC-MPI*XMAM)-Wevap*WAC = 3.22	Acqua da smaltire post-cottura [kg]
ECG	GCH*ETC*RDP = 1.41	Energia termica consumata per cuocere RDP kWh]
ECE	ECH*ETC*RDP = 0.289	Energia elettrica consumata per cuocere RDP kWh]
NSCOD	RDP*1000/Portion = 20	No. scodelle utilizzate
NCL	NSCOD/NCOP = 0.714	No. cicli di lavaggio in lavastoviglie per consumare RDP

Table S1.23
Waste scenario for plastic packaging wastes (PLW).

Products				
Waste specification	Amount	Unit	Category	Comment
SDC -22 Waste scenario plastic wastes	1	kg	Others	

Inputs

Inputs from technosphere: materials/fuels	Amount	Unit	Distrit	SD2 ‹	Min	Max	Comment
Add							

Inputs from technosphere: electricity/heat	Amount	Unit	Dis	S	Comment
Add					

Outputs

Materials and/or waste types separated from waste stream	Material / Waste type	Percentage	Comment
Add			

Waste streams remaining after separation	Percentage	Comment
Waste plastic, mixture {RoW}| treatment of waste plastic, mixture, sanitary landfill | Cut-off, S	6.7 %	
Mixed plastics (waste treatment) {GLO}| recycling of mixed plastics | Cut-off, S	48.7 %	
Waste plastic, mixture {RoW}| treatment of waste plastic, mixture, municipal incineration | Cut-off, S	44.6 %	

Table S1.24
Waste scenario for cardboard and paper packaging wastes (PCW).

Products

Waste specification	Amount	Unit	Category	Comment
SDC -23 Waste scenario Cardboard&paper packagi	1	kg	Others	

Inputs

Inputs from technosphere: materials/fuels	Amount	Unit	Distrit	SD2 (	Min	Max	Comment

Inputs from technosphere: electricity/heat	Amount	Unit	Dis	S	Comment

Outputs

Materials and/or waste types separated from waste stream	Material / Waste type	Percentage	Comment

Waste streams remaining after separation	Percentage	Comment
Waste paperboard {RoW}| treatment of waste paperboard, sanitary landfill | Cut-off, S	5.2 %	
Paper (waste treatment) {GLO}| recycling of paper | Cut-off, S	87.3 %	
Waste paperboard {RoW}| treatment of waste paperboard, municipal incineration | Cut-off, S	7.5 %	

Table S1.25
Waste scenario of non-ferrous metal waste (NFMW).

Products

Waste specification	Amount	Unit	Category	Comment
SDC -24 Waste scenario non-ferrous metal wastes	1	kg	Others	

Inputs

Inputs from technosphere: materials/fuels	Amount	Unit	Distrit	SD2 ‹	Min	Max	Comment

Inputs from technosphere: electricity/heat	Amount	Unit	Dis	S	Comment

Outputs

Materials and/or waste types separated from waste stream	Material / Waste type	Percentage	Comment

Waste streams remaining after separation	Percentage	Comment
Waste aluminium {RoW}| treatment of waste aluminium, sanitary landfill | Cut-off, S	25.4 %	
Aluminium (waste treatment) {GLO}| recycling of aluminium | Cut-off, S	68.1 %	
Scrap copper {Europe without Switzerland}| treatment of scrap copper, municipal incineration | Cut-off, S	6.5 %	

Table S1.26
Disposal scenario of primary packaging material wastes (DS-1pack**).**

Name	Status	Comment
SDC - 25 Disposal Scenario of Primary Packaging	None	

Referring to assembly	Amount	Unit	Comment
SDC - 15 Assembly of Primary Packaging	1	p	

Processes	Amount	Unit	D	M	M	Comment
Municipal waste collection service by 21 metric ton lorry {GLO}| market for municipal waste collection service by 21 metric ton lorry	(11.941/1000)*50 = 0.597	kgkm				TR - Wastes to MSWCS: 50 km

Waste scenarios	Percentage	Comment
SDC -23 Waste scenario Cardboard&paper packaging	55.3 %	
SDC -24 Waste scenario non-ferrous metal wastes	2.8 %	
SDC -22 Waste scenario plastic wastes	41.9 %	

Disassemblies	Percentage	Comment

Reuses	Percentage	Comment

Table S1.27

Disposal scenario of secondary packaging material wastes (DS-2pack**).**

Name	Status	Comment
SDC - 26 Disposal Scenario of Secondary Packaging	None	

Referring to assembly	Amount	Unit	Comment
SDC - 16 Assembly of Secondary Packaging	1	p	

Processes	Amount	Unit	D	M	M	Comment
Municipal waste collection service by 21 metric ton lorry {GLO}| market for municipal waste collection service by 21 metric ton lorry	(308/1000)*50 = 15.4	kgkm				TR - Wastes to MSWCS: 50 km

Waste scenarios	Percentage	Comment
SDC -23 Waste scenario Cardboard&paper packaging	98.7 %	
SDC -22 Waste scenario plastic wastes	1.3 %	

Disassemblies	Percentage	Comment

Reuses	Percentage	Comment

Table S1.28
Disposal scenario of tertiary packaging material wastes (DS-3pack**).**

Name	Status	Comment
SDC - 27 Disposal Scenario of Tertiary Packaging	None	

Referring to assembly	Amount	Unit	Comment
SDC - 17 Assembly of Tertiary Packaging	1	p	

Processes	Amount	Unit	D	M	M	Comment
Municipal waste collection service by 21 metric ton lorry {GLO}| market for municipal waste collection service by 21 metric ton lorry	(PAL*.02/1000)*50 = 0.00	tkm				TR - Wastes to MSWCS: 50 km
Add						

Waste scenarios	Percentage	Comment
SDC -22 Waste scenario plastic wastes	0.2 %	
Add		

Disassemblies	Percentage	Comment
Add		

Reuses	Percentage	Comment
SDC - 29 Semipallet reuse	99.8 %	
Add		

Table S1.29
Disposal scenario of the primary, secondary and tertiary packaging material wastes (DS-1-3pack**).**

Name	Status	Comment
SDC - 28 Disposal Scenario of I-II-III Packaging	None	

Referring to assembly	Amount	Unit	Comment
SDC - 19 Assembly of I-II-III packages	1	p	

Processes	Amount	Unit	D	N	Comment
Municipal waste collection service by 21 metric ton lorry {GLO}| market for municipal waste collection service by 21 metric ton lorry | Cut-off, S	(0.294)*50 = 14.7	kgkm			TR - Paper & Plastic Wastes in a pallet to MSWCS: 5

Waste scenarios	Percentage	Comment
SDC -22 Waste scenario plastic wastes	19.845 %	
SDC -23 Waste scenario Cardboard&paper packaging	79.331 %	
SDC -24 Waste scenario non-ferrous metal wastes	0.824 %	

Disassemblies	Percentage	Comment

Reuses	Percentage	Comment

Table S1.30
Reuse phase of the PP semi-pallet.

Input/output | Parameters

Name	Status	Comment
SDC - 29 Semipallet reuse	None	

Referring to assembly	Amount	Unit	Comment
SDC - 17 Assembly of Tertiary Packaging	1	p	

Processes	Amount	Unit	D	N	Comment
Transport, freight, light commercial vehicle {Europe without Switzerland}| market for transport, freight, light commercial vehicle | Cut-off, S	(PAL*.998)/1000*1	tkm			TR: Sale Points - AP: 150 km

Input/output | **Parameters**

Input parameters	Value	Distribution	SD2 or 2SD	Min	Max	Hide	Comment
PAL	5	Normal	0.5			☐	Mass of semipallet PE [kg]

Table S1.31
Life cycle of dried pulses (LC-DP).

Input/output | Parameters

Name	Status	Comment
SDC - 30 Life cycle of Dried Pulses	None	

Assembly	Amount	Unit	Distributioı	SD2 or 2SD	Min	Max	Comment
SDC - 20 Assembly of Dry Grains	2	p	Undefined				Two 500-g bags

Processes	Amount	Unit	Di:	M	Comment
Transport, freight, light commercial vehicle {Europe without Switzerland}| market for transport, freight, light commercial vehicle | Cut-off, S	(PPDP/MDP*1)*150 = 168	kgkm			TR - Dried Pulses - Points of Sale: 150 km
SDC - 21 Use phase	1	kg	Ur		

Waste/Disposal scenario	Comment

Additional life cycles	Number	Distributioı	SD2 or 2SD	Min	Max	Comment
SDC -31 Life Cycle of I-II-III packaging	1/144 = 0.00694					

Input/output | **Parameters**

Input parameters	Value	Distribution	SD2 or 2SD	Min	Max	Hide	Comment
PPDP	161.42	Undefined				☐	Mass del Semipallet carico [kg]
MDP	144	Undefined				☐	Massa di Legumi secchi nel semipallet [kg]

Table S1.32

Life cycle of the primary, secondary, and tertiary packaging materials (LC-1-3pack).

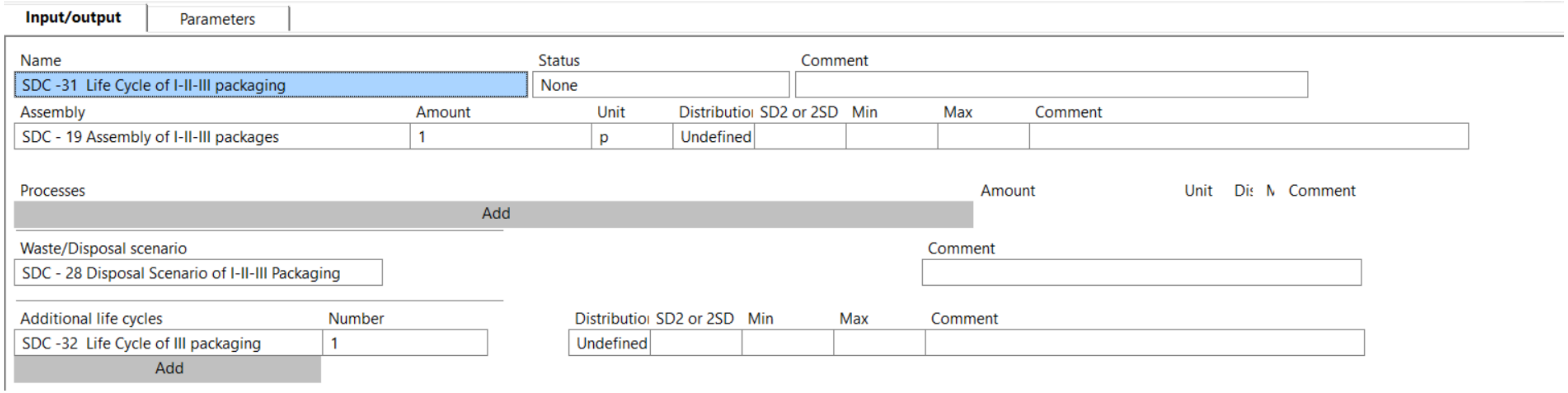

Input/output | Parameters

Name	Status	Comment
SDC -31 Life Cycle of I-II-III packaging	None	

Assembly	Amount	Unit	Distributioi	SD2 or 2SD	Min	Max	Comment
SDC - 19 Assembly of I-II-III packages	1	p	Undefined				

Processes	Amount	Unit	Di:	M	Comment
Add					

Waste/Disposal scenario	Comment
SDC - 28 Disposal Scenario of I-II-III Packaging	

Additional life cycles	Number	Distributioi	SD2 or 2SD	Min	Max	Comment
SDC -32 Life Cycle of III packaging	1	Undefined				
Add						

Table S1.33
Life cycle of the PP semi-pallet (LC-3pack).

Input/output | Parameters

Name	Status	Comment
SDC -32 Life Cycle of III packaging	None	

Assembly	Amount	Unit	Distributio	SD2 or 2SD	Min	Max	Comment
SDC - 17 Assembly of Tertiary Packaging	1	p	Undefined				

Processes	Amount	Unit	Dis	M	Comment
Add					

Waste/Disposal scenario	Comment
SDC - 27 Disposal Scenario of Tertiary Packaging	

Additional life cycles	Number	Distributio	SD2 or 2SD	Min	Max	Comment
Add						

A comprehensive study from cradle-to-grave on the environmental profile of malted legumes

Mauro Moresi and Alessio Cimini

SUPPLEMENTARY MATERIAL S2

Table S2.1 Input/output data for the conventional cultivation of the 3 varieties of legumes examined here (SDC, GPB, LO), as referred to a nominal land area of 1 ha. Air and water emissions were estimated according to the IPCC [45] and EPD® [46] guidelines.

Legume / **Input/Output Parameter**	**SDC**	**GPB**	**OL**	**Unit**
Nominal non-irrigated land used (S)	1	1	1	ha
**Input**				
NPK Fertilizer	100.0	100.0	20.0	kg/(ha yr)
N	20.0	20.0	4.0	kg/(ha yr)
P	8.7	8.7	1.7	kg/(ha yr)
K	16.6	16.6	3.3	kg/(ha yr)
Poultry Manure	200.0	200.0	200.0	kg/(ha yr)
N	8.0	8.0	8.0	kg/(ha yr)
P	3.5	3.5	3.5	kg/(ha yr)
K	6.6	6.6	6.6	kg/(ha yr)
**Output**				
Fresh grain yield	1.71	1.26	1.26	Mg/(ha yr)
Average dry matter in grains harvested	0.88	0.88	0.88	g/g
Harvested annual dry matter grain yield	1.505	1.109	1.109	Mg/(ha yr)
Above ground biomass (AGR)	2.55	2.10	2.10	Mg/(ha yr)
Ratio of above-ground residues dry matter to harvested crop yield	2.1	2.1	2.1	g/g
Ratio of below-ground biomass to above-ground biomass (R_{BG-BIO})	0.19	0.19	0.19	g/g
N content of above-ground residues (N_{AG})	0.008	0.008	0.008	g/g
N content of below-ground residues (N_{BG})	0.008	0.008	0.008	g/g
Fraction of above-ground residues removed ($Frac_{Removed}$)	0			%
Fraction of total area under crop annually renewed ($Frac_{Renew}$)	1			-
Below-ground residues (BG)	770.5	610.2	610.2	kg ss/(ha yr)
Nitrogen in crop residues (F_{CR})	26.57	21.71	21.71	kg N/(ha yr)
Fraction of synthetic N fertilizer volatilized as NH_3 + NO_X ($Frac_{GASF}$)	0.11 (0.02-0.33)			(kg NH_3-N+NO_X-N)/(kg N)
Fraction of organic N fertilizer volatilized as NH_3 + NO ($Frac_{GASM}$)	0.21 (0.0-0.31)			(kg NH_3-N+NO_X-N)/(kg N)
Fraction of all N added lost through leaching off and runoff ($Frac_{LEACH}$)	0.24 (0.01-0.73)			kg N/(kg N)
N_2O-N emissions per unit mass of N fertilizer added (EF_1)	0.01 (0.001-0.018)			kg N_2O-N/(kg N)
N_2O-N emissions per unit mass of NH_3 and NO_X emitted (EF_4)	0.01 (0.002-0.018)			kg N_2O-N/(kg NH_3-N+kg NO_X-N)
N_2O-N emissions per unit mass of N leached off (EF_5)	0.011 (0.0-0.020)			kg N_2O-N/(kg N lisc/dilavato)
Direct N_2O emissions F_{N2ODIR} [=(F_S+F_{CR}) · EF_1 · 44/28]	0.857	0.781	0.530	kg N_2O/(ha yr)
NH_3+NO_X	3.88	3.88	2.12	kg (NH_3-N+NO_X-N)/(ha yr)
$F_{NH3-NOX}$	0.061	0.061	0.033	kg N_2O/(ha yr)

Nitrate emissions in water [NO_3^-]	13.10	11.93	8.09	kg NO_3^--N/(ha yr)
F_{NO3-}	0.226	0.206	0.140	kg N_2O/(ha yr)
Indirect N_2O emissions (F_{N2OIND})	0.287	0.267	0.173	kg N_2O/(ha a)
N_2O emissions from managed soils ($F_{N2O} = F_{N2ODIR} + F_{N2OIND}$)	1.145	1.048	0.703	kg N_2O/(ha a)
P emissions in water [0.05 kg P/ kg P applied] [46]	0.61	0.61	0.26	kg P/(ha a)
NO emissions in air [46]	0.731	0.731	0.178	kg NO-N/(ha a)

Table S2.2

Overall material balance of the fumigation process, cleaning/optical selection of harvested legumes, and storage of cleaned grains shown in Fig. 2, as related to the average yield of harvested fresh grain pulses. All symbols used to identify the various stream flows are given in the Nomenclature section.

**Legume**	**S**	**GRs**	**GR**	**AlP**		**PH_3**	**GRF**	**SP**	**GP**	**RP**	**PO+ER**	**SS**		**GPP**	**H_2O_v**
	ha	kg/ha	kg	Comp.	g	g	kg	kg	kg	kg	kg	No.	kg	kg	kg
SDC	10	1710	17100	85.5	256.5	85.5	17100.2	2137.7	14962.5	769.5	1368.2	15.0	44.9	15007.4	598.5
GPB	8.5	1260	10710	53.6	160.7	53.6	10710.1	1338.9	9371.3	482.0	856.9	9.4	28.1	9399.4	374.85
LO	30	1260	37800	189.0	567.0	189.0	37800.4	12285.4	25515.0	4725.0	7560.4	25.5	76.5	25591.5	1020.6

**Legume**	**GPPD**	**Seeds**	**GPD**	**SGP**	**MZ**	**SSU**	**SSU**	**RPS**
	kg	**kg**	**kg**	**kg**	kg	**No**	kg	kg
SDC	14364.0	1300.0	13064.0	65.3	834.8	0.8	2.5	837.3
GPB	8996.4	850.0	8146.4	40.7	522.7	0.5	1.6	524.3
LO	24494.4	3000.0	21494.4	107.5	4832.5	4.8	14.5	4847.0

Table S2.3

Selling prices (sp) for the cleaned and selected grains of the legumes examined (SDC, GPD, LO) and by-products designated for animal husbandry (MZ) along with the total revenue, and allocation percentage of by-products (ALL_{MZ}). All symbols used are given in the Nomenclature section.

**Legume**	**sp_{GPD}**	**sp_{MZ}**	**MZ**	**GPD**	**Total revenue**	**ALL_{MZ}**
	[€/kg]	[€/kg]	[kg]	[kg]	[€]	[%]
SDC	3.6	0.1	834.8	13064	47113.9	0.18%
GPB	6.0	0.1	522.7	8146.4	48930.7	0.11%
LO	5.0	0.1	4832.5	21494.4	107955.2	0.45%

Table S2.4
Percentage fraction of the process and packaging materials discarded, as collected in the dry legume factory examined in this work.

**Processing and Packaging Materials discarded**	**Discarded Fraction (%)**
PE super-sacks	0
Dry legumes	0.5
Primary packaging	
PP bags	2.5
Paper label	0.5
Cardbord collar	0.5
Brass rivets	0.5
Secondary packaging	
Cartons (CAW)	1.0
Carton labels	0.3
Scotch tape	0.3
Tertiary packaging	
Damaged PP semi-pallet	0.2
Pallet adhesive label	0.5
Stretch & shrink film	0.5

Table S2.5

Overall material balance of the packaging process of cleaned pulse grains and its waste management process., as referred to the block diagram in Fig. 3. All symbols used to identify the various stream flows are given in the Nomenclature section.

**Legume**	**GPD**	**SGP**	**Bags**	**PP**	**N_2**	**CAV**	**RO**	**ET**	**SPP**	**SCAV**	**SRO**	**SET**
	[kg]	[kg]	No.	[kg]	[kg]	[kg]	[kg]	[kg]	[kg]	[kg]	[kg]	[kg]
SDC	13064.0	65.3	25997.4	133.2	130.0	151.5	8.7	21.1	3.2	0.754	0.043	12.7
GPB	8146.4	40.7	16211.3	83.1	81.1	94.5	5.4	13.2	2.0	0.470	0.027	7.9
LO	21494.4	107.5	42773.9	219.2	213.9	249.3	14.3	34.7	5.3	1.240	0.071	20.9

**Legume**	**LCI**	**CA**		**SCA**	**EC**	**SEC**	**SC**	**SSC**
	[kg]	No.	[kg]	[kg]	[kg]	[kg]	[kg]	[kg]
SDC	13426.5	2166.4	660.8	6.5	4.3	0.01	8.7	0.03
GPB	8372.5	1350.9	412.1	4.1	2.7	0.01	5.4	0.02
LO	22090.8	3564.5	1087.2	10.8	7.2	0.02	14.3	0.04

**Legume**	**LCII**	**PAL**		**SPAL**	**EP**	**SEP**	**FP**	**SFP**	**LCIII**
	[kg]	no.	[kg]	[kg]	[kg]	[kg]	[kg]	[kg]	[kg]
SDC	14093.8	90.3	452.2	0.9	0.6	0.003	26.1	0.13	14571.6
GPB	8788.5	56.3	282.0	0.6	0.4	0.002	16.3	0.08	9086.5
LO	23188.7	148.5	744.1	1.5	0.9	0.005	42.9	0.21	23974.9

**Legume**	**RPL'**	**RCC'**	**RM'**	**MZ**	**PO+E**
	[kg]	[kg]	[kg]	[kg]	[kg]
SDC	49.2	20.0	0.043	834.8	1368.2
GPB	30.8	12.5	0.027	522.7	856.9
LO	83.6	32.9	0.071	4832.5	7560.4

Table S2.6

Overall material balance of the malting, cleaning, dehulling and packaging processes of cleaned pulse grains and their waste management process, as referred to the block diagrams in Figures S1 and 3. All symbols used to identify the various stream flows are given in the Nomenclature section.

**Legume**	**GPD**	**MP**	**PCR**	**MPC**
	[kg]	[kg]	[kg]	[kg]
SDC	13064.0	13064.0	1920.4	11143.6
GPB	8146.4	8146.4	1197.5	6948.9
LO	21494.4	21494.4	3159.7	18334.7

**Legume**	**SMP**	**PP**		**N_2**	**CAV**	**RO**	**ET**	**SPP**	**SCAV**	**SRO**	**SET**	**MPCI**
	[kg]	No.	[kg]	[kg]	[kg]	[kg]	[kg]	[kg]	[kg]	[kg]	[kg]	[kg]
SDC	55.7	22175.7	113.7	110.9	129.3	7.4	18.0	2.8	0.643	0.037	10.8	11452.8
GPB	34.7	13828.3	70.9	69.1	80.6	4.6	11.2	1.7	0.401	0.023	6.8	7141.7
LO	91.7	36486.1	187.0	182.4	212.7	12.2	29.6	4.6	1.058	0.061	17.8	18843.5

**Legume**	**CA**		**SCA**	**EC**	**SEC**	**SC**	**SSC**	**MPCII**	**PAL**		**SPAL**	**EP**	**SEP**	**FP**	**SFP**	**MPCIII**
	No.	[kg]	[kg]	[kg]	[kg]	[kg]	[kg]	[kg]	No.	[kg]	[kg]	[kg]	[kg]	[kg]	[kg]	[kg]
SDC	954.4	291.1	2.9	1.9	0.01	3.8	0.01	11746.8	77.0	385.8	0.8	0.5	0.002	22.2	0.11	12154.4
GPB	595.1	181.5	1.8	1.2	0.00	2.4	0.01	7325.0	48.0	240.6	0.5	0.3	0.001	13.9	0.07	7579.2
LO	1570.3	479.0	4.7	3.2	0.01	6.3	0.02	19327.1	126.7	634.7	1.3	0.8	0.004	36.6	0.18	19997.8

**Legume**	**RPL’**	**RCC’**	**RM’**	**MZ**	**PO+E**
	[kg]	[kg]	[kg]	[kg]	[kg]
SDC	48.6	14.4	0.037	2745.6	1368.2
GPB	30.4	9.0	0.023	1714.2	856.9
LO	82.6	23.6	0.061	7976.4	7560.4

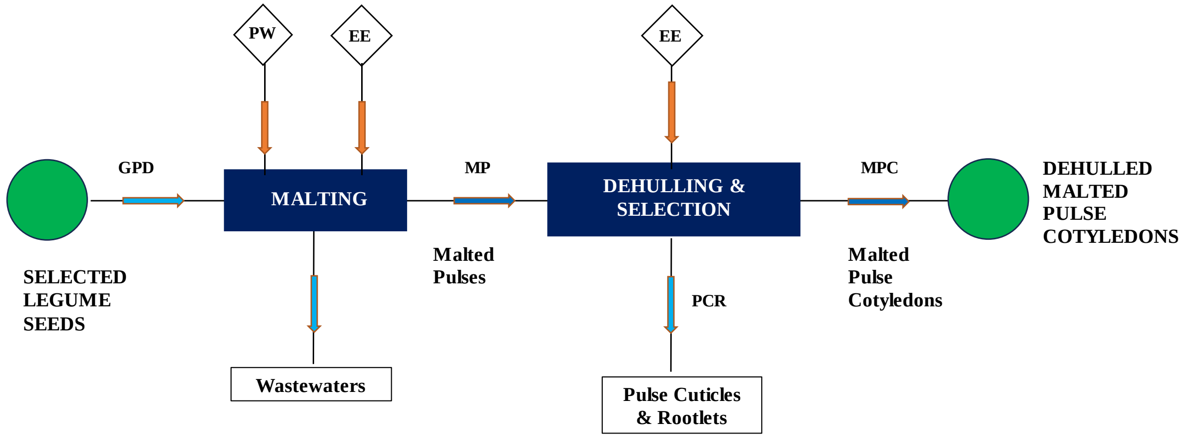

Figure S2.1. Block diagram of the malting, dehulling and selection process of selected legume seeds. For all symbols refer to the Nomenclature section.
